# The Effects of Nocturnal Hypoxemia on Cognitive Performance in Andean Highlanders

**DOI:** 10.1089/ham.2024.0077

**Published:** 2024-12-10

**Authors:** Elizabeth V. Young, Matea A. Djokic, Erica C. Heinrich, Traci Marin, Cecilia Anza-Ramirez, Jeremy E. Orr, Dillon Gilbertson, Pamela N. DeYoung, Gustavo Vizcardo-Galindo, Rómulo Figueroa-Mujica, Francisco C. Villafuerte, Atul Malhotra, Tatum S. Simonson

**Affiliations:** ^1^ Division of Pulmonary, Critical Care, Sleep Medicine, and Physiology, Department of Medicine, University of California, San Diego, California, USA.; ^2^ Department of Ecology and Evolutionary Biology, University of California, Irvine, California, USA.; ^3^ Division of Biomedical Sciences, School of Medicine, University of California Riverside, Riverside, California, USA.; ^4^ Laboratorio de Fisiología Comparada/Fisiología del Transporte de Oxígen, Facultad de Ciencias y Filosofía, Universidad Peruana Cayetano Heredia, Lima, Peru.

**Keywords:** Andean Highlanders, cognition, high-altitude physiology, nocturnal hypoxemia, sleep-disordered breathing

## Abstract

Young, Elizabeth V., Matea A. Djokic, Erica C. Heinrich, Traci Marin, Cecilia Anza-Ramirez, Jeremy E. Orr, Dillon Gilbertson, Pamela N. DeYoung, Gustavo Vizcardo-Galindo, Rómulo Figueroa-Mujica, Francisco C. Villafuerte, Atul Malhotra, and Tatum S. Simonson. The effects of nocturnal hypoxemia on cognitive performance in andean highlanders. *High Alt Med Biol*. 26:347–354, 2025.

**Background:**

Many Andean highlanders exposed to chronic hypoxemia are susceptible to excessive erythrocytosis (EE) and chronic mountain sickness (CMS). Nocturnal hypoxemia is more marked than diurnal hypoxemia and includes sustained and intermittent components. The potential for cognitive impairments related to nocturnal hypoxemia in this population has not been extensively studied, but improved understanding may provide opportunities for the prevention of long-term effects of EE and CMS.

**Methods:**

To examine this relationship, 48 participants residing permanently at 4,340 m completed an overnight sleep study and a battery of cognitive function tests that examined a broad range of cognitive domains.

**Results:**

Greater nocturnal hypoxemia was associated with longer reaction times on balloon Analogue Risk Task (BART) (*p* < 0.01) and Emotion Recognition Test (ERT) (*p* < 0.01). Longer completion times of Trail Making Task were also associated with increased nocturnal hypoxemia (*p* = 0.03). Increased hematocrit was similarly associated with longer reaction times on the ERT (*p* = 0.01) and the BART (*p* = 0.01).

**Conclusion:**

Overall, our results showed that increased nocturnal hypoxemia and higher hematocrit were associated with impairments in cognitive performance in individuals residing permanently at high altitude.

## Introduction

Chronic hypoxemia is a major effect of high altitude due to decreased barometric pressure and the lower partial pressure of inspired oxygen (O_2_). Hypoxemia can lead to cerebral hypoxia and cause impairment across multiple cognitive domains including reaction time (Frost et al., 2021), attention, motor function (Griva et al., 2017), and working memory (De Aquino Lemos et al., 2012), with complex cognitive processes being particularly susceptible (Taylor et al., 2015). At high altitude, hypoxemia experienced during sleep has been shown to have a greater effect on cognition than hypoxemia while awake (Frost et al., 2021), and some of these impairments improve with supplemental O_2_ ((Gerard et al., 2000); Van der Post et al., 2002; Heinrich et al., 2019a).

High altitude exposure causes several sleep disruptions including sleep-disordered breathing (SDB), which is marked by periodic breathing with intermittent O_2_ desaturation (Bloch et al., 2010; Ainslie et al., 2013), reduced REM (rapid eye movement) sleep (Tseng et al., 2015), and sleep fragmentation (Bloch et al., 2015). Nocturnal O_2_ enrichment mitigates many of these disruptions and improves daytime O_2_ saturation, suggesting nocturnal hypoxemia in particular plays a key role in the consequences of exposure to high altitude (Luks et al., 1998).

Permanent residence at high altitude is associated with negative effects on cognitive performance, but in contrast to the aforementioned studies on lowlanders traveling to altitude, little is understood about the relationship between hypoxemia during sleep and cognition in high-altitude residents (Su et al., 2024). Andean highlanders have a greater prevalence of SDB than a matched sample of lowland individuals (Pham et al., 2017a), and some Andeans also demonstrate decreased ventilatory sensitivity to O_2_ and carbon dioxide (CO_2_) compared with lowlanders (Julian et al., 2013; Beall and Strohl, 2021), which may result in more severe and prolonged desaturation events during sleep (Azarbarzin et al., 2019; Heinrich et al., 2020). Increased intermittent hypoxemia during sleep in highlanders is known to have major cardiometabolic consequences (Pham et al., 2017b) with likely negative impacts on cognition and memory (Kiratli et al., 2010; Shanjun et al., 2020; Zhen et al., 2022; Pase et al., 2023).

SDB and nocturnal hypoxemia are more severe in Andean highlanders with excessive erythrocytosis (EE) (Rexhaj et al., 2016; Heinrich et al., 2020), defined as hematocrit (Hct) >63% in men and >57% in women, a hallmark of chronic mountain sickness (CMS) (Villafuerte et al., 2022). EE affects more than 30% of highlanders living above 4,000 m by their mid-50s (Villafuerte et al., 2022) and may be associated with ventilatory control as many highlanders with EE have lower ventilatory sensitivities to CO_2_ relative to individuals without EE (Julian et al., 2013). Higher Hct may suggest more exposure to hypoxemia over time, with notable implications for health and cognition (Corante et al., 2018; Shanjun et al., 2020). Subjective poor sleep quality and EE are associated with modest impairment in short-term memory and processing speed on cognitive testing in Andean highlanders (Heinrich et al., 2019b), and a higher CMS score has been linked to impaired cognitive performance (Shanjun et al., 2020). The relationship between cognitive performance and quantitative nocturnal hypoxemia in this population has not been examined.

This pilot study aimed to explore the relationship between nocturnal hypoxemia and cognitive performance as well as Hct and cognitive performance in Andean men and women. We hypothesized that low nocturnal O_2_ saturation and high Hct would be separately associated with reduced accuracy and slower response times on a cognitive test battery examining multiple measures of cognition, including emotion recognition, risk assessment, attention, reflex speed, motor speed, and cognitive flexibility.

## Materials and Methods

### Ethical approval

This study was approved by the University of California, San Diego Human Research Protection Program 140235. Participants were informed of the purpose and risks of the study and provided written consent in their native language (Spanish).

### Participants

Men and women aged 18–65 years, who self-reported to be lifelong residents of Cerro de Pasco, Peru, with at least three previous generations of high altitude (>2,500 m) Andean ancestry were recruited by flyers and word-of-mouth in Cerro de Pasco, Peru (∼4,340 m).

Exclusion criteria included self-reported history of pulmonary, cardiovascular, or renal disease, pregnancy, active smoking, regular alcohol use, recent blood transfusion or phlebotomy, or travel to <4,000 m altitude in the previous 6 months.

During the screening visit, participants were assessed for CMS based on Qinghai CMS score criteria. These criteria consider Hct or hemoglobin (with different criteria based on sex) concentration at altitude of residence and assess for presence and severity of symptoms including breathlessness, palpitations, sleep disturbance, cyanosis, dilation of veins, paresthesia, headache, and tinnitus (León-Velarde et al., 2005). Baseline physical characteristics were assessed, including height, weight, resting heart rate, and oxygen saturation (SpO_2_) during wakefulness. Participants with abnormal basic medical examination, EKG (electrocardiogram), or spirometry were excluded.

Participants were asked not to consume alcohol, caffeine, coca leaves, or tea for 8 hours before testing and to avoid anti-inflammatory medications for 24 hours before testing.

Hct values were collected from peripheral venous blood samples. Blood was collected using standard phlebotomy procedures into heparin-coated glass capillary tubes, centrifuged, and measured.

### Sleep studies

Forty-eight participants completed a single overnight sleep study, 44 of which produced adequate oximetry recordings. Participants were monitored with a limited-channel polysomnogram (Respironics Alice PDx, Murrysville, PA, USA). Recordings consisted of nasal pressure, finger pulse oximetry, thoracic and abdominal effort bands, right and left electro-oculogram, two-channel electroencephalogram using electrodes C3 and C4, and chin electromyogram. Participants also wore a WatchPAT device, which measures pulse oximetry and fingertip peripheral arterial tonometry (Itamar Medical, Caesarea, Israel). Studies were scored by a registered polysomnographic sleep technologist using American Academy of Sleep Medicine criteria for scoring and Chicago criteria for events (Berry et al., 2012). The sleep technologist reviewed individual SpO_2_ tracings from pulse oximetry measurements, verified pulse oximetry data against WatchPAT data, and removed artifacts appropriately.

Outcomes of interest included nocturnal SpO_2_, time spent with SpO_2_ <80%, nadir nocturnal SpO_2_, apnea–hypopnea index (AHI), and oxygen desaturation index (ODI). An SpO_2_ cutoff of 80% was selected in order to adequately capture desaturation events, as most participants had an awake SpO_2_ of less than 90%. ODI was defined as desaturations ≥4% from baseline lasting at least 10 seconds measured per hour.

### Cognitive function testing

Upon waking, participants performed a cognitive test battery of well-established tests used in previous high-altitude studies (Rimoldi et al., 2016; Frost et al., 2021). In addition, several tests were used from the *Cognition* computerized test battery developed to evaluate cognitive performance during spaceflight (Basner et al., 2015). The written cognitive function test was administered in Spanish in a quiet room. The digital tests were administered in English with the assistance of a Spanish interpreter. The digital tests have not been formally validated in a Spanish-speaking population but have been translated and used by native Spanish speakers (Padrón et al., 2015; Sanabria et al., 2019). The written test has been validated in a Spanish-speaking population (Arango-Lasprilla et al., 2015). All tests were administered by the same two trained research personnel. Each test was administered only once to each participant to minimize any learning effect.

Digital Tests:

The **Emotion Recognition Test (ERT)**, part of the *Cognition* test package, assesses the ability to identify emotions in facial expressions (Montagne et al., 2007; Basner et al., 2015). A series of 30 portraits of actors of varying age, gender, and ethnicity enacting emotions in a randomized order were displayed on an iPad. Participants were given a selection of five emotions to attribute to each face: happy, sad, anger, fear, or no emotion. Scores were based on the percentage of correct responses and reaction times.

The **Balloon Analogue Risk Task (BART)**, part of the *Cognition* test package, assesses risk-taking behavior (Basner et al., 2015). iPads were used to display a digital balloon worth “$1” with the option to collect the money or pump the balloon. Participants were awarded $1 for a balloon pump, but the balloon inflated with the risk of popping and losing the money. Participants completed 30 trials with randomization of the number of pumps before the balloon pops. Data were collected on the amount of money collected and reaction time to select an option.

The **Psychomotor Vigilance Test (PVT)** is part of the *Cognition* test package and measures sustained attention, reflex speed, and behavioral alertness (Basner et al., 2015). *Cognition* uses a validated 3-minute version of the PVT (Basner et al., 2011). Participants monitored a red box in the center of an iPad screen. When the box changed colors, a millisecond timer became visible and participants were asked to tap the screen as fast as possible. Mean reaction times and lapses were recorded.

Written test:

The **Trail Making Test (TMT)** assesses motor speed and cognitive flexibility (Bowie and Harvey, 2006). Participants completed TMTa, which involved connecting consecutive numbers on a page in one continuous line (1, 2, 3…), and TMTb, in which alternating letters and numbers must be connected in consecutive order (1, A, 2, B…). Participants were evaluated on time to complete the task.

### Statistical analysis

Data were collected and managed using REDCap electronic data capture tools hosted at the University of California, San Diego (Harris et al., 2019). All statistical analyses were performed in R version 4.3.1. Shapiro–Wilk normality tests were performed and found the data to be non-normally distributed; therefore, Spearman correlations were performed using the *rcorr* function (Hollander and Wolfe, 1973; Press et al., 1988) housed in the *Hmisc* package. Paired data were row matched and Spearman correlations were calculated with the default parameters whereby correlations were performed on the nn-missing parameters. Correlations with a *p* value <0.05 were considered significant. For each step of the analysis, participants who were missing values were excluded.

## Results

### Participants

An equal number of men (*N* = 24) and women (*N* = 24) were included in the study. Participant demographics have been previously described by Heinrich et al., 2020. For this study, an additional woman participant was excluded from the analysis because of incomplete cognitive testing data for a total of 22 men and 22 women. There were several demographic parameters that differed significantly between men and women participants, including age, CMS score, Hct, and heart rate. The ages of the men ranged from 25 to 65, and the ages of the women ranged from 19 to 63. Four of the men participants (17%) and none of the women had a CMS score that exceeded 5, diagnostic of CMS. Using cutoffs of Hct >63% for men and Hct >56% for women, 4 men (17%) and 2 women (8%) met criteria for EE.

Education level of participants was not recorded. A variety of occupations were represented, but the most common were housewife (15), construction/maintenance/electrical work (9), and student (6). To our knowledge, no participants were currently employed by mining industries.

There was no significant effect of age or daytime SpO_2_ on the outcome parameters discussed below.

### Sleep study outcomes

Sleep study data with a focus on SDB and nocturnal hypoxemia are presented in Table 1. Detailed analysis of mean and nadir SpO_2_ has been previously described by Heinrich et al., 2020. Mean measures of SDB and nocturnal hypoxemia did not significantly differ between men and women. Prevalence of obstructive sleep apnea defined as AHI > 5 was high among participants, with 17 of the men (77%) and 13 of the women (59%) meeting criteria. CMS score was not associated with AHI or ODI. AHI was not significantly associated with any of the cognitive outcomes examined.

**Table 1.** Summary of Sleep Study Outcomes of Study Participants

Variable	Men (n = 22)	Women (n = 22)	p *value*
Study duration (hours)	7.35 ± 0.90	7.67 ± 1.06	0.28
Total sleep duration (hours)	6.42 ± 0.89	6.81 ± 1.10	0.20
Stage 1 sleep duration (hours)	0.74 ± 0.29	0.65 ± 0.36	0.35
Stage 2 sleep duration (hours)	3.00 ± 0.81	3.04 ± 0.90	0.87
Stage 3 sleep duration (hours)	0.30 ± 0.32	0.64 ± 0.60	0.02
REM sleep duration (hours)	1.08 ± 0.51	1.05 ± 0.31	0.85
AHI	22.51 ± 16.84	18.02 ± 17.78	0.42
ODI	29.38 ± 32.62	16.25 ± 10.78	0.08
% Time asleep SpO_2_ < 80% (%)	41.84 ± 29.80	41.94 ± 38.44	0.99

AHI, apnea–hypopnea index; ODI, oxygen desaturation index.

### Nocturnal hypoxemia and ERT and BART reaction times

**FIG. 1.**
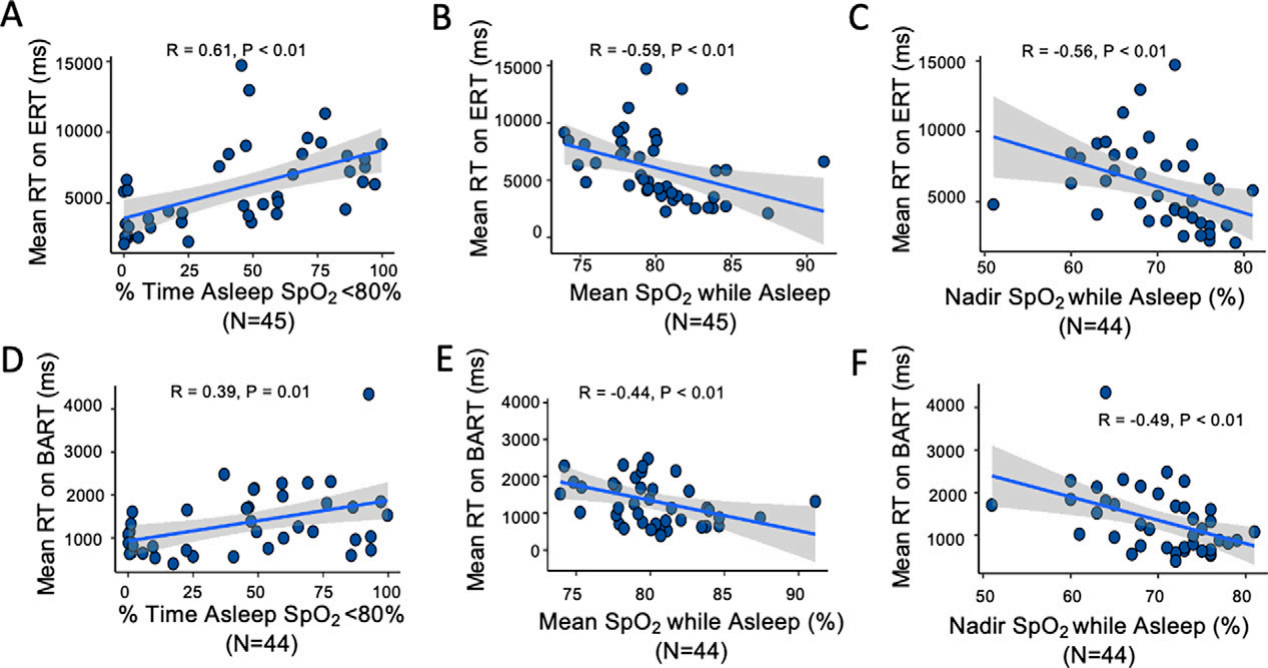
Nocturnal hypoxemia and reaction times. **(A–C)** Spearman correlation of percentage of sleep time with SpO_2_ <80%, mean SpO_2_ while sleeping, and nadir SpO_2_ while sleeping with mean reaction time (RT) on the Emotion Recognition Test (ERT). **(D–F)** Spearman correlation of percentage of sleep time with SpO_2_ <80%, mean SpO_2_ while sleeping, and nadir SpO_2_ saturation with the Balloon Analogue Risk Task (BART). The shaded area indicates the 95% confidence interval for the best-fit regression line, which is shown by the solid black line.

Mean reaction times on the ERT (*p* < 0.01) were slower in participants with greater percentage of sleep time spent with SpO_2_ < 80% (Fig. 1A), lower mean SpO_2_ while asleep (Fig. 1B), and lower nadir SpO_2_ (Fig. 1C). The same results were observed for reaction times using the BART (*p* < 0.01) (Fig. 1D–F).

### Nocturnal hypoxemia and performance on the ERT

Spearman analysis did not reveal any significant relationships between ERT performance (as measured by percentage of correct responses) and nocturnal hypoxemia. Specifically, the percentage of correct responses was not associated with percentage of sleep time spent with SpO_2_ <80% (*p* = 0.57, *R* = −0.09), nadir SpO_2_ (*p* = 0.17, *R* = 0.22), or mean SpO_2_ while asleep (*p* = 0.53, *R* = 0.10).

### Nocturnal hypoxemia and performance on the BART

Spearman analysis did not reveal any significant relationships between BART performance (as measured by the amount of reward collected) and nocturnal hypoxemia. In particular, reward collected on the BART was not associated with the percentage of sleep time spent with SpO_2_ <80% (*p* = 0.7, *R* = −0.06), nadir SpO_2_ (*p* = 0.11, *R* = 0.25), or mean SpO_2_ while asleep (*p* = 0.21, *R* = 0.2).

### Nocturnal hypoxemia and the TMTs

**FIG. 2.**
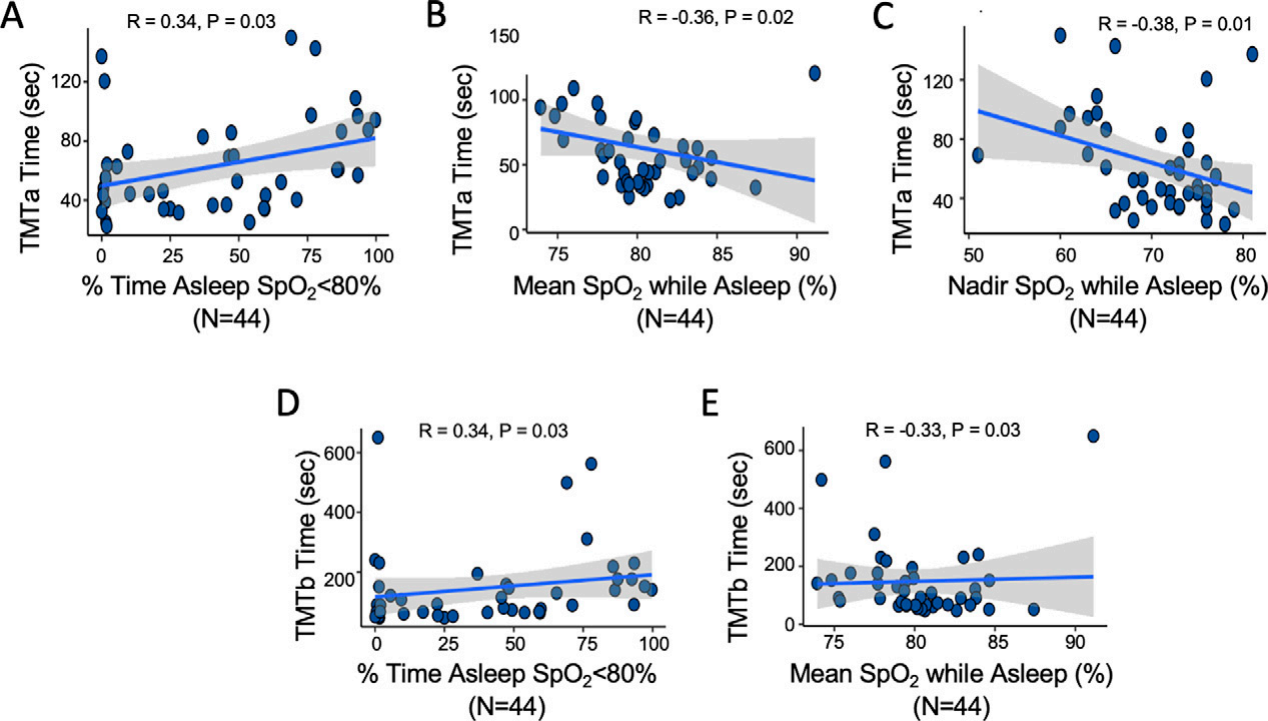
Nocturnal hypoxemia and Trail Making Test (TMT) completion times. **(A–C)** Spearman correlation pf percentage of sleep time with SpO_2_ <80%, mean SpO_2_ while sleeping and nadir SpO_2_ while sleeping with time to complete the Trail Making Test version A (TMTa). **(D–E)** Spearman correlation of percentage of sleep time with SpO_2_ <80% and mean SpO_2_ while sleeping with time to complete the Trail Making Test version B (TMTb). The shaded area indicates the 95% confidence interval for the best-fit regression line, which is shown by the solid black line.

TMTa completion time was longer in participants with a greater percentage of sleep time spent with SpO_2_ <80% (Fig. 2A), lower mean SpO_2_ while asleep (Fig. 2B), and lower nadir SpO_2_ (Fig. 2C). Similar results were observed for TMTb with the exception of nadir SpO_2_ (Fig. 2D–E).

### Nocturnal hypoxemia and PVT performance

Spearman analysis of PVT performance, as measured by reaction time and number of lapses, did not reveal any significant relationships between PVT performance and nocturnal hypoxemia. PVT mean reaction time was not associated with the percentage of sleep time spent with SpO_2_ <80% (*p* = 0.13, *R* = 0.24), nadir SpO_2_ (*p* = 0.17, *R* = −0.22), or mean SpO_2_ while asleep (*p* = 0.55, *R* = −0.1). PVT lapses were similarly not associated with the percentage of sleep time spent with SpO_2_ <80% (*p* = 0.55, *R* = 0.1), nadir SpO_2_ (*p* = 0.59, *R* = −0.09), or mean SpO_2_ while asleep (*p* = 0.8, *R* = 0.04).

### Hct and cognitive test outcomes

**FIG. 3.**
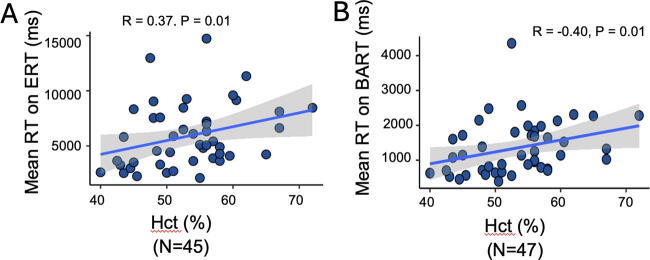
Hematocrit (Hct) and reaction times. **(A–B)** Spearman correlation of Hct percentage with the reaction time on the ERT and BART, respectively. The shaded area indicates the 95% confidence interval for the best-fit regression line, which is shown by the solid black line. BART, Balloon Analogue Risk Task; ERT, Emotion Recognition Test.

Higher Hct was correlated with longer mean reaction times on the ERT and BART (Fig. 3). Hct was not associated with the percentage of correct responses on the ERT (*p* = 0.41, *R* = −0.13), reward collected on the BART (*p* = 0.47, *R* = −0.11), TMTa completion time (*p* = 0.28, *R* = 0.17), TMTb completion time (*p* = 0.33, *R* = 0.15), PVT mean reaction time (*p* = 0.56, *R* = 0.09), or PVT lapses (*p* = 0.73, *R* = 0.05).

## Discussion

This study examined the relationship between nocturnal hypoxemia, Hct, and cognitive function in Andean highlanders. Our results showed a relationship between hypoxemia during sleep and several measures of cognitive performance. In particular, more severe nocturnal hypoxemia was associated with longer complex reaction times (CRT) and time to complete complex tasks (Figs. 1 and 2). Higher Hct was similarly associated with these outcomes (Fig. 3). Overall, these results suggest greater levels of nocturnal hypoxemia and higher Hct are associated with impaired cognitive performance in lifelong high-altitude residents.

Higher Hct and greater nocturnal hypoxemia were significantly associated with longer reaction times on ERT and BART, complex tasks that measure risk taking and emotion recognition (Fig. 1). Simple reaction time as measured by the PVT was not associated with measures of nocturnal hypoxemia. CRT, as evaluated by tasks such as ERT and BART, has been the most consistently observed impairment during acute acclimatization to altitude, particularly in controlled laboratory settings (Bliemsrieder et al., 2022). Nocturnal hypoxemia has been shown to independently predict increased reaction times in the laboratory setting (van der Post et al., 2002). Increased CRT may be a protective adjustment attributable to a delay in stimulus identification required to correctly respond to complex tasks such as the ERT and BART (Fig. 1), rather than a motor delay, especially given simple reaction time is generally not as affected (Yan, 2014). These impairments have also been observed in individuals with acute mountain sickness, but CRT may improve with acclimatization (Pun et al., 2018; Pun et al., 2019). Data examining this process in populations permanently residing at altitude are very limited, but our results suggest persistent delays in CRT despite long-term exposure.

Impairments in emotion recognition, as measured by ERT performance, were not associated with greater hypoxemia during sleep. To our knowledge, this is the first study to investigate emotion recognition in a population permanently residing at high altitude. There is some evidence that there is a relationship between greater nocturnal hypoxemia and impaired emotion recognition during acute acclimatization to high altitude (Heinrich et al., 2019a).

Nocturnal hypoxemia was not associated with the award amount collected on the BART, a gambling task and measure of risk taking. The concurrent longer reaction times suggest this group possibly spent more time considering each action; however, this did not appear to impact performance. There is evidence in prior work that acute normobaric hypoxia exposure results in less aversion to losses in risk-taking tasks (Pighin et al., 2014). The lifetime exposure to hypobaric hypoxia and further intermittent hypoxemia during sleep may explain the difference between these outcomes.

In Tibetans, chronic altitude exposure has a higher risk of long-term effects on cognition compared with acute acclimatization, with findings likely applicable to other permanent high-altitude populations (Yan et al., 2011). The mechanism of these persistent impairments is unclear though it has been independently linked to both subjectively diminished sleep quality and higher Hct (Kong et al., 2011). There are several proposed mechanisms regarding structural changes that may explain these findings, such as cerebral hypometabolism as a protective measure against chronic hypoxemia, which may be accentuated by nocturnal desaturations (Hochachka et al., 1994; Farhat and Weber, 2021). Another possibility is that EE may cause structural brain damage through the physical consequences of hyperviscosity (Villafuerte et al., 2022), which is supported by our data that demonstrate an association between higher Hct and reduced cognitive performance. There is evidence of genetic adaptations in different high-altitude populations that may mitigate such effects of chronic hypoxemia (Simonson, 2015). This range of proposed mechanisms behind impaired cognition in high-altitude residents illustrates the complexity of the relationship between hypoxemia and EE. EE is a known consequence of chronic hypoxemia; yet not all individuals living permanently at high altitude develop EE and CMS, as illustrated in our study population. A minority of the cohort had findings consistent with EE; however, there was a clear relationship between both nocturnal hypoxemia and Hct with cognitive outcomes, raising the question of the extent to which each contributes. CMS score was not associated with measures of SDB, which does suggest that SDB may independently impact cognition. Further analysis controlling for hypoxemia and Hct in turn was limited by sample size, but clearly indicates an intriguing direction for further work.

This study included an equal number of men and women participants, though there were several differences between the two groups. Women were younger, with lower Hct and CMS scores. At baseline, women have lower Hct than men, and the Qinghai criteria for CMS account for this (León-Velarde et al., 2005). CMS is less common in premenopausal women, attributed in part to the protective nature of progesterone which increases alveolar ventilation, and in part due to the natural phlebotomy of menstruation (Villafuerte and Corante, 2016). However, this relationship is not currently well understood and warrants future detailed research. Despite this, in our study, there was no significant difference in the sleep outcomes of interest including rates of SDB between men and women. The associations between nocturnal hypoxemia and cognition also did not significantly differ when separated by sex.

Our sample did capture the full range between the inclusion ages of 18 and 65. Adjusting for age did not change the outcome parameters in this analysis, though CMS is more common with advancing age in populations permanently dwelling at high altitude (Villafuerte and Corante, 2016), and particularly more common in postmenopausal women compared to premenopausal women (León-Velarde et al., 1997). Age-related cognitive decline also could have an impact on cognitive performance. Our study may not have been sufficiently powered to detect significant differences attributable to age and sex. Future work with more substantial sample sizes to illuminate these relationships would be of great interest.

This study was limited by several factors, particularly the use of this single cohort, the small size of which limits generalizability. Health conditions were self-reported, and we were limited in our ability to fully capture environmental exposures, such as biofuel use and arsenic exposure, both of which are present in the area of residence of the study population. In addition, we conducted a battery of neurocognitive tests and multiple comparisons based on our *a priori* review of the literature and our previous research in individuals acclimatizing to high altitude (Heinrich et al., 2019a), specifically with regard to the impact of nocturnal hypoxemia. The chosen cognitive tests were also potentially limiting as the TMT was the only one used that has been validated in Spanish speakers. The effects this may have had on the results are unclear. We advocate for rigorous follow-up studies to confirm or refute these new findings. As this study was observational, interventions such as O_2_, hemodilution, positive airway pressure, or pharmacologic variables were not examined. Despite these limitations, we do believe that these findings are novel and illustrate clear targets for future research in this area.

## Conclusion

Our findings demonstrate a clear relationship between nocturnal hypoxemia and higher Hct and impairment across several cognitive domains, particularly CRT, motor speed, and cognitive flexibility in chronic high-altitude dwellers. While chronic hypoxemia experienced at altitude has been previously understood to have negative impacts on cognition, our findings demonstrate the role that increased sustained and intermittent hypoxemia during sleep has in this population.
